# Response and oil degradation activities of a northeast Atlantic bacterial community to biogenic and synthetic surfactants

**DOI:** 10.1186/s40168-021-01143-5

**Published:** 2021-09-21

**Authors:** Christina N. Nikolova, Umer Zeeshan Ijaz, Clayton Magill, Sara Kleindienst, Samantha B. Joye, Tony Gutierrez

**Affiliations:** 1 Institute of Mechanical, Process and Energy Engineering, School of Engineering and Physical Sciences, Heriot-Watt University, Edinburgh, EH14 4AS UK; 2 School of Engineering, University of Glasgow, Glasgow, G12 8LT UK; 3 Institute for GeoEnergy Engineering, School of Energy, Geoscience, Infrastructure and Society, The Lyell Centre, Edinburgh, EH14 4AS UK; 4 Center for Applied Geosciences, Eberhard Karls University of Tübingen, Tübingen, Germany; 5 Department of Marine Sciences, The University of Georgia, Athens, GA USA

**Keywords:** Dispersant, Biosurfactant, Rhamnolipid, Marine environment, Crude oil, Hydrocarbons, Biodegradation, Faroe-Shetland Channel

## Abstract

**Background:**

Biosurfactants are naturally derived products that play a similar role to synthetic dispersants in oil spill response but are easily biodegradable and less toxic. Using a combination of analytical chemistry, 16S rRNA amplicon sequencing and simulation-based approaches, this study investigated the microbial community dynamics, ecological drivers, functional diversity and robustness, and oil biodegradation potential of a northeast Atlantic marine microbial community to crude oil when exposed to rhamnolipid or synthetic dispersant Finasol OSR52.

**Results:**

Psychrophilic *Colwellia* and *Oleispira* dominated the community in both the rhamnolipid and Finasol OSR52 treatments initially but later community structure across treatments diverged significantly: *Rhodobacteraceae* and *Vibrio* dominated the Finasol-amended treatment, whereas *Colwellia*, *Oleispira*, and later *Cycloclasticus* and *Alcanivorax*, dominated the rhamnolipid-amended treatment. Key aromatic hydrocarbon-degrading bacteria, like *Cycloclasticus,* was not observed in the Finasol treatment but it was abundant in the oil-only and rhamnolipid-amended treatments. Overall, Finasol had a significant negative impact on the community diversity, weakened the taxa-functional robustness of the community, and caused a stronger environmental filtering, more so than oil-only and rhamnolipid-amended oil treatments. Rhamnolipid-amended and oil-only treatments had the highest functional diversity, however, the overall oil biodegradation was greater in the Finasol treatment, but aromatic biodegradation was highest in the rhamnolipid treatment.

**Conclusion:**

Overall, the natural marine microbial community in the northeast Atlantic responded differently to crude oil dispersed with either synthetic or biogenic surfactants over time, but oil degradation was more enhanced by the synthetic dispersant. Collectively, our results advance the understanding of how rhamnolipid biosurfactants and synthetic dispersant Finasol affect the natural marine microbial community in the FSC, supporting their potential application in oil spills.

**Video abstract**

**Supplementary Information:**

The online version contains supplementary material available at 10.1186/s40168-021-01143-5.

## Background

Extensive tracking of the microbial response to crude oil contamination in the ocean after the Deepwater Horizon (DWH) oil spill in the Gulf of Mexico in 2010 provided an unprecedented view into feedbacks between environmental chemical signatures and microbial community evolution [1]. During this historic spill, approximately 700,000 tonnes (4.9 million barrels) of Louisiana light sweet crude oil was discharged into the Gulf from a blown-out wellhead at a depth of ~ 1500 m. Because of the scale and nature of the oil spill, synthetic dispersants were the primary response tool employed [2]. The decision to use synthetic dispersants during a marine oil spill is driven largely by the desire to keep oil from reaching sensitive coastlines—often the primary goal of dispersant application. This unprecedented dispersant application involved approximately 7 million litres of the synthetic dispersants Corexit 9500 and 9527 to sea surface oil slicks and directly at the discharging wellhead at the seabed [3]. Prior to the DWH incident, limited knowledge of the effects of synthetic dispersants use on open ocean microbial communities was available. As a consequence, questions were raised about the response of autochthonous populations of hydrocarbon-degrading (hydrocarbonoclastic) bacteria—key players in oil biodegradation—to these dispersants, and the need to identify the impact of dispersants on oil bioremediation was highlighted.

Following the DWH incident, a number of studies investigated the effects of Corexit on natural microbial communities; some studies also reported the response of oil biodegradation rates. Corexit appeared to inhibit natural microbial oil biodegradation in some cases, possibly due to the toxicity by one or more of the dispersant ingredients and/or because some microbes that responded to dispersants (e.g., *Colwellia* spp.) preferred to metabolize dispersant constituents more than oil [4, 5]. Some studies have reported that Corexit, and other synthetic dispersants, stimulated oil biodegradation by increasing its bioavailability to microorganisms [6–8]. Although the main components of synthetic dispersants are food-grade surfactants, including Tween 80 and Span 80, other components are hydrocarbon-based solvents that could confer toxicological impacts, while others are unknown because they are proprietary knowledge. Furthermore, a commonly used surfactant in dispersant formulations is dioctyl sodium sulfosuccinate (DOSS), a known toxin [9], that persists in the environment for months [10] to years in cold (deep sea) environments [11]. It is, therefore, logical to search for natural-based solutions to reduce the toxic footprint conferred by some of the ingredients, such as DOSS, in synthetic chemical dispersant formulations. Hopeful candidates for natural, non-toxic, and biodegradable substitutes for synthetic chemical dispersants are microbial biosurfactants [12].

Many bacterial species produce biosurfactants [13] that serve a similar purpose as synthetic dispersants, namely to reduce the surface and interfacial tension between oil droplets and seawater and increase the rate of oil biodegradation [14]. The most commonly studied biosurfactant producer is *Pseudomonas aeruginosa,* a ubiquitous bacterial species that grows on a wide range of hydrocarbon and non-hydrocarbon substrates and is known for its production of the glycolipid surfactant rhamnolipid, which is known for its excellent surface-active properties (reduction of the surface tension of water from 72 mN m^− 1^ to less than 30 mN m^−1^) and ability to facilitate the formation of stable petrol and diesel emulsions [15]. Rhamnolipids have been shown to be effective in dispersing crude oil and enhancing its biodegradation by pre-selected bacterial consortia [16], some of which containing oil-degrading strains of *Ochrobactrum* sp. and *Brevibacillus* sp. [17]. However, studies comparing the effects of synthetic and bio-based surfactants on indigenous marine microbial communities are rare. A study compared the effects of the biosurfactant surfactin produced by *Bacillus* sp. strain H2O-1, to a synthetic dispersant, Ultrasperse II, using a natural marine microbial community, including its biodegradation of crude oil [18]. The surfactin enriched for hydrocarbonoclastic bacteria more so than the synthetic dispersant, but no difference in oil biodegradation across treatments was observed. Similar results were found in a more recent study in which rhamnolipid, trehalose, and sophorolipid biosurfactants were compared to three commercial dispersants in oiled microcosms with marine coastal water. Although the biosurfactants caused differential microbial responses, the rate of alkane biodegradation was similar to the microcosms amended with dispersants [19].

In this study, we investigated whether the presence of a rhamnolipid and the synthetic dispersant Finasol OSR52, which is stockpiled worldwide for use in oil spill response, would result in a distinct shift in the taxonomic composition of a natural marine microbial community from the Faroe-Shetland Channel (FSC) and which taxa would be more likely responsible for the compositional shifts over time. The FSC is a subarctic region located on the UK Continental Shelf west of the Shetland Islands. The region is distinctive as it has a 20-year history of oil exploration and production, with some fields located in deep waters, up to 1500 m (e.g., Lagavulin) [20]. The FSC has complex and dynamic physical circulation characterized by mixing of distinct water masses [21] and the area is remote, cold, and characterized by rough weather conditions for the majority of the year, meaning that an oil spill response there would be challenging. No major oil spills in the deep waters of this region have been documented that would draw direct comparisons with the DWH event in relation to microbial response and fate of crude oil. However, an ocean general circulation model with particle tracking algorithm demonstrated that oil spilled on the sea surface (< 200 m depth) would likely advect northwards and potentially reach the Arctic regions of eastern Greenland, Svalbard and into the Barents Sea within a year of the release, whereas oil releases in deeper waters (> 600 m) were predicted more likely to flow westwards and reach southern Greenland, the Labrador Sea and on towards Newfoundland [22]. In addition, the FSC hosts important biological diversity, such as deep-sea sponges, cold-water coral communities, and a vibrant commercial fishing industry [21] which could become negatively impacted by a major oil spill, especially in the event of a subsea blowout. Revealing which taxa are the key players driving the shifts in the microbial communities would help to better understand and predict the microbial dynamics during oil biodegradation in situ, and therefore, support the oil spill response decision-making process in the region. Based on this knowledge, ecological null models can be built to estimate the benefits or disadvantages of using synthetic chemical dispersants or biosurfactants in oil spill response [23]. Furthermore, we investigated how the synthetic dispersant and biosurfactant affected the microbial diversity, community assembly, and the taxa-functional relationship (i.e., the link between a community’s taxonomic composition and its functional profile) and robustness (i.e., the degree at which a shift in a community’s taxonomical composition will impact its functional capacities) [24] over time. For determining the taxa-function relationship, we utilized the PICRUSt2 tool [25], and for the taxa-function robustness, we implemented the method of Eng and Borenstein [24]. Determining a community’s functional robustness can further help estimate the functional impact of dispersant application during oil spills to gauge how susceptible the microbial communities are to disruption of function due the presence of the synthetic chemical dispersant compared with the biosurfactant. Lastly, we performed Gas Chromatography-Flame ionization detection coupled with mass spectrometry (GC-FID/MS) to track the crude oil biodegradation in the experimental microcosms.

## Materials and methods

### Field sampling and water accommodated fractions preparation

**Fig. 1:**
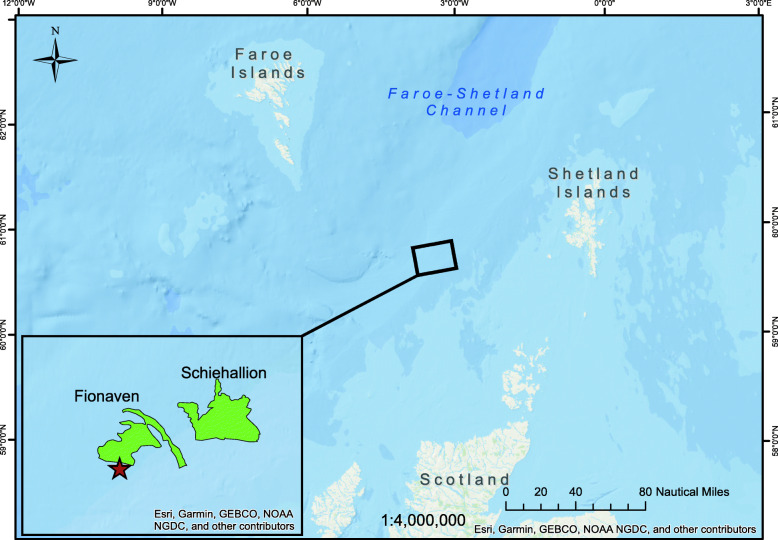
Map of the sampling site location (red star) and nearby oil-producing fields (green) in the Faroe-Shetland Channel. The map was created with ArcGIS Map software ver.10.6.1 (ESRI, USA) and freely available data from Oil & Gas UK

Surface seawater was collected on May 2018 from 3 m depth in the Faroe-Shetland Channel (FSC) (60°16.36′ N, 04°20.60′ W; Fig. 1), which is a subarctic, deep-water region of the northeast Atlantic characterized by an active oil and gas industry (Supplementary Methods). Immediately after sampling, the seawater was transferred onboard to 10 L carboys and stored at 4 °C, and used within 2 days after returning to the laboratory at Heriot-Watt University for the set-up of the experimental microcosms.

To assess the changes in the microbial community structure and dynamics during enrichment with crude oil and in the presence of either the synthetic dispersant Finasol, or the biosurfactant rhamnolipid, three main water accommodated fractions (WAFs) were prepared in acetone-rinsed, acid-washed and autoclaved 2 L glass aspirator bottles according to established methods [4, 26], though with some modifications. For preparation of the WAFs, the collected seawater was filtered (0.22 μm; Millipore) in order to avoid the possibility of bacterial growth during the preparation of the WAFs. The first WAF contained seawater and crude oil only and is hereon referred to as WAF. A Chemically Enhanced WAF (CEWAF) was prepared with seawater, crude oil and addition of Finasol OSR-52 (Total Fluides, Paris, France) at a dispersant-to-oil (DOR) ratio of 1:20. Biosurfactant Enhanced WAF (BEWAF) was prepared with seawater, crude oil and rhamnolipid (at least 90% monorhamnolipids produced by *P. aeruginosa*) at the same DOR as in the CEWAF. All three WAFs contained the same volume of filter-sterilized seawater (1560 ml) and Schiehallion crude oil (120 ml; API 25°; BP) which also originates from the FSC. Each of the three main WAFs (WAF, CEWAF, BEWAF) were prepared by combining the prescribed quantities of seawater, crude oil and synthetic dispersant or biosurfactant in the aspirator bottles and leaving the solutions to mix on a rotary magnetic stirrer (140 rpm; 10 °C) for up to 48 h. In addition, two control WAFs were set up in the same way to assess the microbial community response to the dispersant or biosurfactant alone and in the absence of the crude oil. One of these contained only seawater and Finasol (SWD); the other contained seawater and the biosurfactant rhamnolipid (SWBS). After mixing for 48 h, the three WAFs containing crude oil were allowed to stand undisturbed for 1 h to allow for any bulk undispersed oil to settle to the surface. Small buoyant oil droplets, however, remained suspended in the aqueous phase. The aqueous phase from each of the WAF mixtures was then carefully collected from the bottom outlet of the bottles, avoiding any of the bulk undispersed surface oil, and this material was used to set up of the microcosms.

### Setup and sampling of microcosm treatments

Five microcosm treatments were set up in acetone-rinsed, acid-washed and autoclaved 0.5-L glass bottles in triplicates. Each treatment contained 66 ml of aliquoted WAF mixture (WAF, CEWAF, BEWAF, SWD, or SWBS) and unfiltered seawater to a total volume of 300 ml, leaving 200 ml of head space to ensure aerobic conditions. In addition, an untreated control comprising seawater with no other additions (SW) was setup and run in parallel. All bottle treatments were placed on a roller table to maintain constant gentle mixing (15 rpm) at approximately 9.7 °C (in situ temperature at the time of sampling) for 28 days in darkness. At the beginning of these incubations (day 0) and then subsequently thereafter at days 3, 7, 14, and 28, each treatment was sub-sampled for total microbial cell count and for DNA extraction (10 ml), following the method described below. To analyze for changes in the hydrocarbon composition of the oil due to biodegradation, additional replicates of the treatments that contained the oil (i.e. the WAF, BEWAF, and CEWAF treatments) were prepared (in triplicate) in identically the same way as described above (Supplementary Methods).

### DNA extraction and barcoded amplicon sequencing

DNA was extracted according to the method of a previous study [27] which utilizes chemical cell lysis with potassium xanthogenate buffer. DNA extracts were resuspended in 20 µl of 1 mM TE buffer and stored at -20 °C for Illumina barcoded-amplicon sequencing. A two-step amplification procedure (Supplementary Methods) was used to amplify the 16S rRNA gene in order to minimize heteroduplex formation in mixed-template reactions [28]. The purified PCR reactions were pooled together and then sent for paired-end Illumina MiSeq sequencing (Illumina 2 × 250 v2 kit) at the Edinburgh Genomics Facility (University of Edinburgh, UK).

### Bioinformatic and statistical analysis

The resulting 16S rRNA gene sequences (7,703,409 pair-end reads) were processed with the open-source bioinformatics pipeline QIIME2 [29]. Initially, sequences were demultiplexed and quality-filtered using the DADA2 algorithm as a QIIME plugin [30]. DADA2 implements a quality-aware correcting model on amplicon data that denoises, removes chimeras and residual PhiX reads, dereplicates DNA reads, and calls amplicon sequence variants (ASVs) [31]. The quality-filtered sequences were then aligned to the reference alignment database SILVA SSU Ref NR release v132 [32]. PICRUSt2 algorithm as a QIIME plugin [25] was used on the 16S rRNA gene sequences to predict the functional abundance and diversity of the microbial community (Supplementary Information). After the bioinformatics steps, we obtained 3412 ASVs for *n* = 91 samples with summary statistics of reads as follows: [1st quantile: 72,593, median: 83,007, mean: 85,704, 3rd quantile: 102,888, max: 144,269], on which we performed the statistical analyses.

Statistical analyses of the ASVs (alpha and beta diversity, subset and regression analyses, functional abundance and diversity, and taxa-function robustness) were performed using the statistical software programme R-Studio v3.6.3 [33] and further described in detail in Supplementary Methods. The R scripts used to generate the analyses are available at http://userweb.eng.gla.ac.uk/umer.ijaz/bioinformatics/ecological.html and as part of R’s microbiomeSeq package http://www.github.com/umerijaz/microbiomeSeq [24].

## Results

### Bacterial diversity

**Fig. 2:**
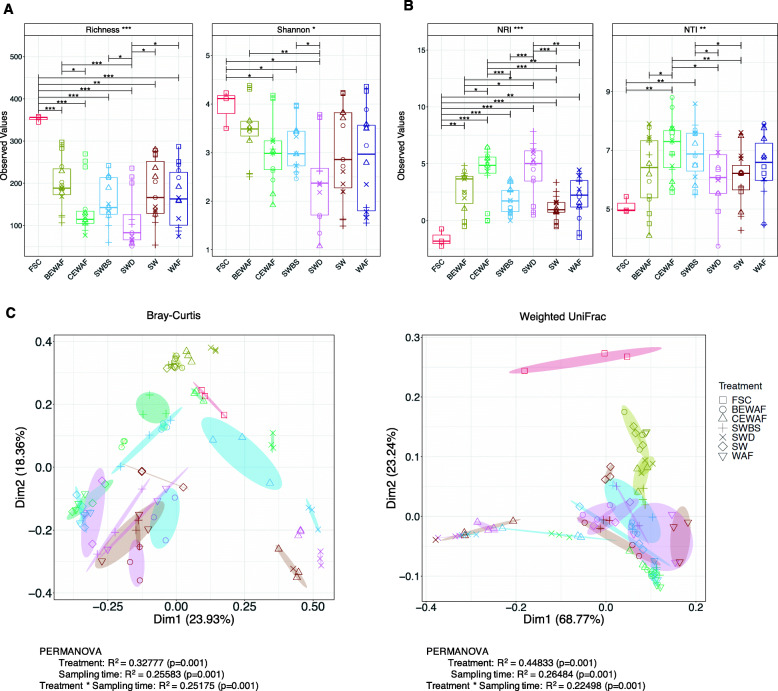
**A** Overall alpha diversity indices of ASVs and **B** net relatedness index (NRI) and nearest-taxon index (NTI). Statistically different treatments (pair-wise ANOVA) are connected by bracket and the level of significance is shown with *(*p* < 0.05), **(*p* < 0.01), or ***(*p* < 0.001). Colors represent treatments and shapes the incubation time (square—day 0, plus—day 3, cross—day 7, circle—day 14, and triangle—day 28). **C** Principal coordinate analysis (PCoA) using Bray-Curtis, unweighted Unifrac, and weighted Unifrac distance matrices. Ellipses represent 95% confidence interval of the standard error of the ordination points of a given grouping. Results from PERMANOVA test for each distance matrix are shown underneath each plot. Colors in **B** represent sampling time (red—in situ seawater at time of collection, olive green—day 0, green—day 3, blue—day 7, pink—day 14, and brown—day 28). FSC is the in-situ baseline microbial community, WAF—seawater and oil only, BEWAF—seawater, crude oil and biosurfactant, CEWAF—seawater, crude oil and synthetic dispersant, SW—seawater only, SWBS—seawater and biosurfactant, and SWD—seawater and synthetic dispersant

Alpha diversity indices, species richness, and Shannon index were calculated across treatments and time. Species richness was highest in the BEWAF treatments and significantly different from Finasol-amended treatments which had the lowest diversity (Fig. 2A). Temporal changes across treatments revealed that the richness was the lowest on day 3 and after that gradually increased for all treatments, albeit with some small differences. Principle coordinates analysis (PCoA) plot revealed distinct clustering of treatments based on Bray-Curtis dissimilarities (Fig. 2C). Taking into account weighted UniFrac distance measure, all treatments clustered close to each other and even overlapped, indicating phylogenetic similarity between the treatments. PERMANOVA confirmed that treatment and incubation time (*p* = 0.001) were significant factors contributing to the beta diversity variance, with treatment explaining up to 45% of the variability and time up to 26% (Fig. 2C). Furthermore, Local Contribution to Beta Diversity (LCBD) analysis showed that diversity varied with time in all treatments but by the end of the incubation period, the community assembly in the CEWAF and SWD treatments were markedly different from the average community structure (Supplementary Figure S1).

Next, we performed subset regression analysis on one-dimensional realisation of the microbiome (alpha and beta diversity) to further understand which of the treatments and/or incubation time points specifically caused an increase or decrease in the microbiome properties. The subset regressions confirmed that the presence of Finasol in the treatments had a significant negative effect on richness and Shannon entropy, but positive on the beta diversity as indicated by LCBD (i.e., caused distinct clustering of samples). In contrast, the inclusion of rhamnolipid led to increase only in Shannon entropy and NRI and had an insignificant influence on the rest of the diversity measures (Supplementary Figure S2).

### Ecological drivers of microbial communities

Ecological processes responsible for changes in microbial communities in each treatment over time were determined by NTI and NRI. All treatments had NTI values that were significantly greater than +2 (*p* < 0.05), indicating strong clustering driven by deterministic environmental filtering (Fig. 2B). The addition of crude oil, either by itself or in combination with Finasol or rhamnolipid, determined the microbial community structure promoting co-existence of closely related and ecologically similar taxa. As expected, the environmental setting had no effect on the community structure in the in situ FSC community as indicated by negative NRI value (Fig. 2B). The relative influence of environmental filtering on the microbial community composition varied over time, but overall it was strongest in the Finasol-amended treatments CEWAF and SWD.

### Bacterial community composition

**Fig. 3:**
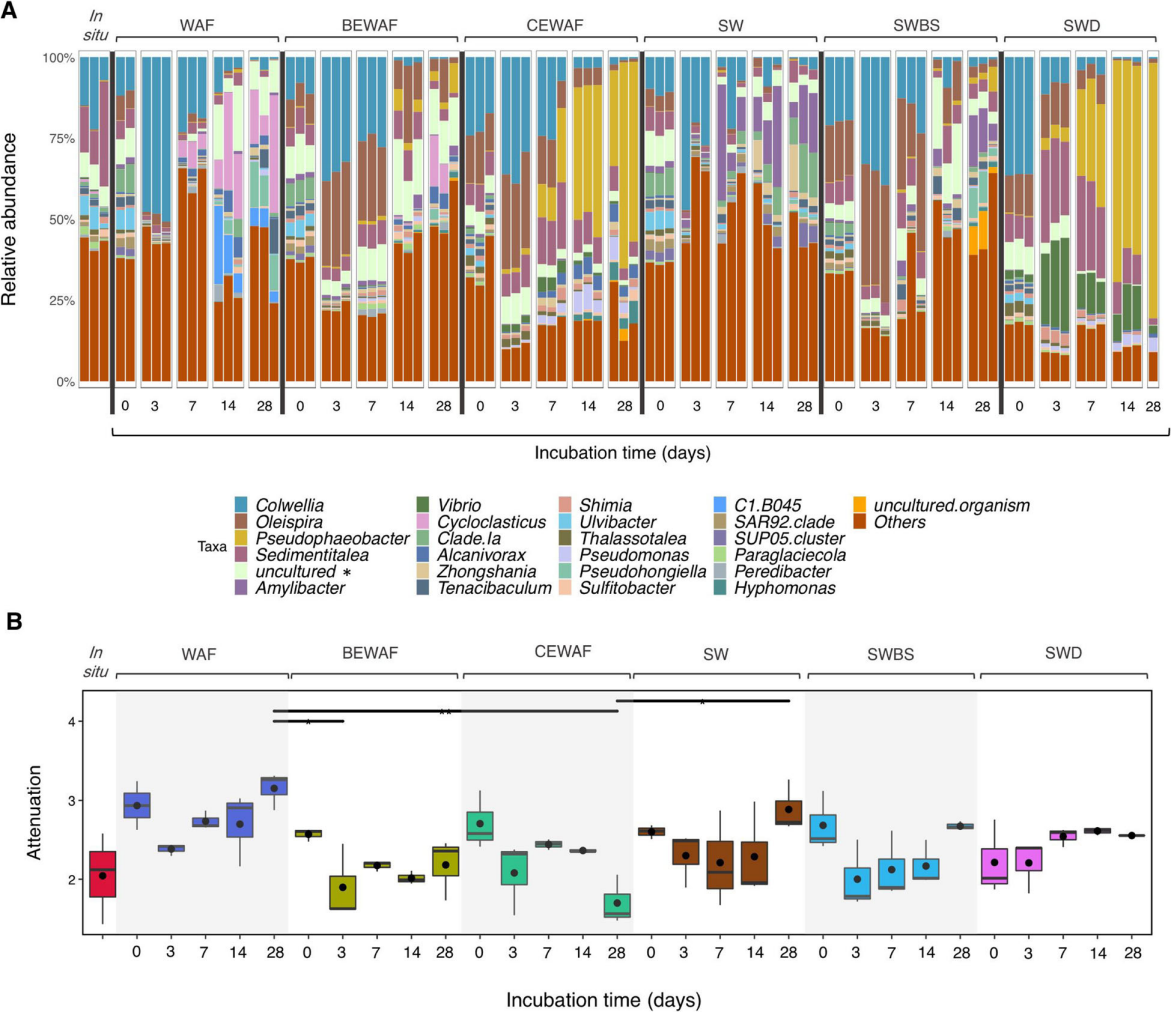
Taxonomic composition of microbial communities. **A** Relative abundance of top 25 most abundant taxa shown to genus level. Treatments at different incubation times are shown as independent triplicates. **B** Taxa-function robustness as expressed as attenuation values, where in situ is baseline microbial community at time of seawater sampling (FSC); WAF—seawater and oil only, BEWAF—seawater, crude oil, and biosurfactant; CEWAF—seawater, crude oil, and synthetic dispersant; SW—seawater only; SWBS—seawater and biosurfactant; and SWD—seawater and synthetic dispersant. In **A** * represents uncultured bacteria from the *Micavibrionaceae* family. SWD had one replicate on day 28 and WAF had two replicates on day 0

Members of the Proteobacteria dominated the taxonomic profiles over the 28-day microcosm incubations, ranging between 60 and 98%. In contrast, the Bacteroidetes decreased from ~ 40% (initially) to almost undetectable by the end of the experiment. Other phyla were present at < 5% relative abundance. At day 0, all treatments showed similar community composition, comparable to the in situ community. Community profiles were dominated at the family level by *Colwelliaceae*, *Saccharospirillaceae*, *Rhodobacteracea*, and *Micavibrionaceae* (> 40%; Fig. 3A). Further details about the relative abundance of the top 25 most abundant taxa in all treatments over time are available in Supplementary File 1.

The initial abundance of *Colwellia* varied significantly across treatments. At day 0, *Colwellia* abundance ranged from 15% in the seawater control (SW) to 11% (WAF, BEWAF) to > 35% in the dispersant only control (SWD) and CEWAF treatments. In the SWD treatment, the abundance of *Colwellia* rapidly decreased to 9%, then to 5%, and then 1% on days 3, 7, and 28, respectively. The abundance of *Oleispira* increased to 28% in BEWAF and CEWAF treatments but was negligibly abundant in the oil-only treatment (Fig. 3A). By day 7, members of uncultured *Micavibrionaceae* increased in both the BEWAF and WAF treatments, peaking on day 14 (18% and 14%, respectively). On day 14 the community profiles of the WAF and BEWAF treatments were quite distinct, with *Colwellia* having markedly decreased in abundance in both treatments to 6% and 2%, respectively. Across all treatments *Cycloclasticus* and *Alcanivorax* were rare initially (< 1%) but had increased by up to 20% by day 14 in the WAF treatment and these abundances were maintained on day 28. In the BEWAF, *Cycloclasticus* and *Alcanivorax* abundances remained below 1% until day 14 but increased to 8% and 4% by day 28, respectively; these taxa were not detected in the SW control. Oil degraders, except *Colwellia*, were not enriched in the SW treatment. Similar to the BEWAF and WAF treatments, on day 3 the CEWAF treatment was dominated by *Colwellia* (35%), *Oleispira* (28%), *Sedimentitalea* (8%), and uncultured members of the *Micavibrionaceae* (9%). *Pseudophaeobacter* and *Sedimentitalea* (family *Rhodobacteracea*) were exclusively enriched in the Finasol-ammended treatements (CEWAF and SWD) by the end of incubation. *Alcanivorax* increased in the CEWAF treatment from < 1% in the early stages of incubation to 5% by the end.

A strong enrichment in *Vibrio* was observed only in the SWD treatment, with abundance increasing from 2% at day 0 to 26% by day 3, followed by a gradual decrease to 12% (day 14), and then to 2.4% (day 28). In the CEWAF treatment, for comparison, *Vibrio* became only slightly enriched (1–4%) throughout the incubation period. *Pseudomonas* was observed mainly in the CEWAF and SWD treatments where its abundance increased from < 1% on day 3, to 6% on day 14 in the CEWAF treatment, and 3% in SWD. In the CEWAF treatment, *Cycloclasticus* was absent.

### Taxa-function robustness

To provide a direct and quantitative comparison of taxa-function robustness differences between treatment communities, we defined the attenuation values for each treatment over time based on the taxa-function response curves. Higher attenuation drives smaller functional shifts and thus higher robustness. Overall, the functional robustness varied temporarily between treatments (Fig. 3B). Generally, robustness decreased slightly on day 3 in all treatments, but by the end of the incubation period it increased in all treatments except the CEWAF treatment. In fact, the attenuation of these communities further revealed a clear but insignificant difference between the main oil-amended treatments, with the CEWAF treatment having the lowest attenuation (1.70) compared to the BEWAF (2.18) and WAF (3.14) treatments at the end of the incubation period.

**Fig. 4:**
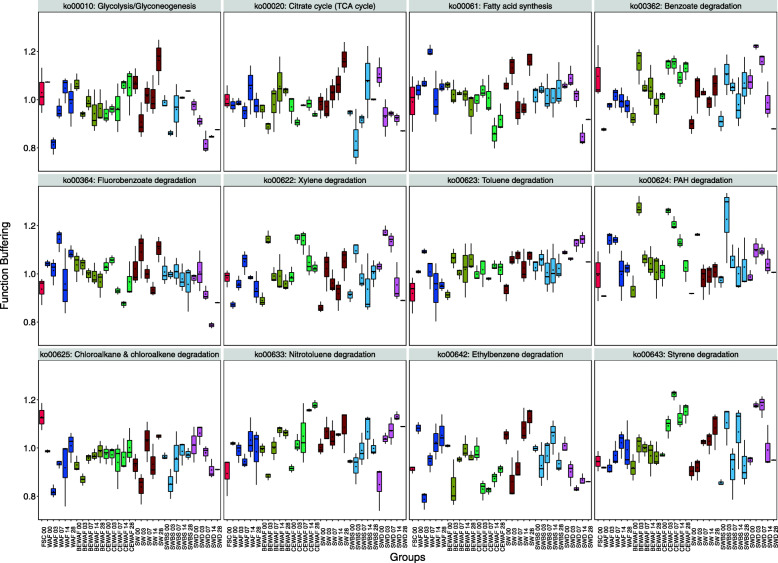
Attenuation values of individual KEGG pathways across all treatments and time. FSC is the in situ baseline microbial community; WAF—seawater and oil only; BEWAF—seawater, crude oil, and biosurfactant; CEWAF—seawater, crude oil, and synthetic dispersant; SW—seawater only; SWBS—seawater and biosurfactant; and SWD—seawater and synthetic dispersant

Given the variation in overall taxa-function robustness observed above, we next examined whether robustness also varied across different functions and whether such function-specific robustness is consistent across treatments. For this, we compared the attenuations of each function between treatments, this time analyzing functions at the pathway level (e.g., hydrocarbon degradation and biosurfactant biosynthesis pathways were of specific interest). The glycolysis and gluconeogenesis pathway (associated with biosurfactant biosynthesis; ko00010), nitrotoluene degradation (ko00633), and styrene degradation (ko00643) functions were less robust in the CEWAF treatment compared to the WAF (*p* = 0.003) and BEWAF treatments by the end of the incubation period (Fig. 4). In contrast, fluorobenzoate (ko00364) and ethylbenzene (ko00642) degradation pathways were significantly more robust in the CEWAF treatment than in the WAF treatment (*p* = 0.046 and *p* = 0.007, respectively) on day 28.

### Key taxa representing major shift in the communities

In order to identify key taxa representing major shifts in the communities across the different treatments, we performed differential abundance analysis with DESeq2 with adjusted *p* value significance cut-off of 0.05 and log 2-fold change (Supplementary File 2). Common oil-degraders belonging to the genera *Marinobacter*, *Oleispira*, and *Pseudomonas* were enriched in all treatments. *Vibrio*, *Oleiphilus*, and *Glaciecola* were enriched exclusively in CEWAF, while *Alcanivorax*, *Colwellia*, and *Thalassotalea* (of the family *Colwelliaceae*) were enriched in both CEWAF and WAF. *Cycloclasticus*, *Pseudohongiella*, and *Acinetobacter* were enriched in the BEWAF and WAF treatments, whereas *Alteromonas*, *Moritella*, and *Paraglaciecola* were enriched exclusively in the BEWAF.

**Fig. 5:**
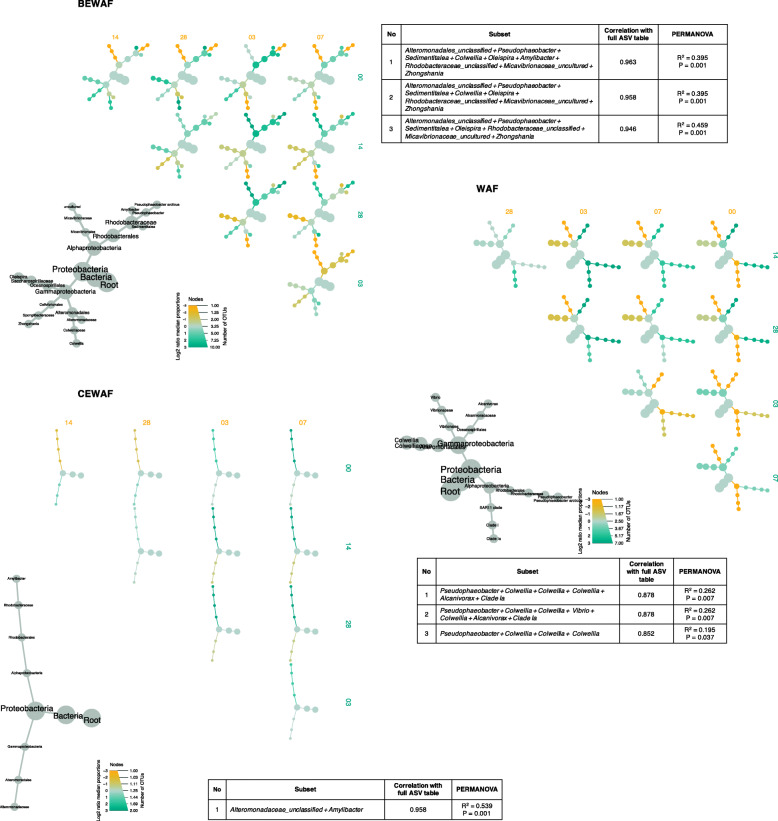
Differential heat trees showing the key differential taxa (DESeq2; using Wilcoxon *p* value test adjusted with multiple comparison) in treatments BEWAF (seawater, crude oil, and biosurfactant), WAF (seawater and oil only), and CEWAF (seawater, crude oil, and synthetic dispersant). Subset analysis was performed to identify the subset of significant ASVs causing the major change in beta diversity in these treatments. The top 3 (where possible) subsets with the highest correlation with the full ASV table considering Bray-Curtis distance (PERMANOVA) are listed for each treatment. The grey trees are taxonomic key for the smaller unlabelled coloured trees. The colour of each taxon represents the log-10 ratio of median proportions of reads observed in each treatment. The size of tree nodes shows the number of ASVs (note: labelled as OTUs) present in each treatment

Next, we considered a subset analysis which determined the minimum set of significant ASVs that can statistically explain the observed variation in community composition for each treatment over time. The subset analysis procedure calculates pair-wise Bray-Curtis distances between samples using all the ASVs in the abundance table. It then permutes through the combination of ASVs until a minimal subset of ASVs is found, in which the beta diversity is conserved against the full ASV table. We used differential heat trees to showcase how members of the highest correlated subsets for each treatment changed their abundances over time. The resulting reduced-order subsets correlated highly with the full table by preserving the beta diversity between samples (Fig. 5). In the BEWAF treatment, *Alteromonadaceae*, *Pseudophaeobacter*, members of the *Rhodobacteraceae* family (*Amylibacter* and *Sedimentitalea*), *Colwellia*, *Oleispira*, and *Micavibrionaceae* significantly drove the shifts in community dynamics over time (*R*^2^ = 0.395, *p* = 0.001), similarly to the seawater control (SW) (Supplementary Figure S3). In the WAF treatment, the taxa driving the observed shifts in community structure over time (*R*^2^ = 0.262, *p* = 0.007) are *Oleispira* and *Alcanivorax*, and putative oil-degraders *Colwellia* and *Pseudophaeobacter*. In the CEWAF treatment, only two taxa, unclassified members of the *Alteromonadaceae* family and *Amylibacter*, drove the diversity shift with 0.96 correlation with the full ASV abundance data (*R*^2^ = 0.539, *p* = 0.001).

### Predicted functional diversity and abundance

The 16S rRNA metagenomic data was used to predict the functional potential of oil-contaminated microbial communities by PICRUSt2 analysis, which identified 10,543 KEGG orthologs (KOs) across all samples. The predicted richness of KO was the highest in the BEWAF treatment, and statistically different from the CEWAF and SWD treatments on days 3 and 7 compared to the rest of the treatments during the same period (Supplementary Figure S4A). By days 14 and 28, the functional richness in the BEWAF and WAF treatments was very similar and close to the maximum number of predicted KO. The SWD treatment displayed the lowest diversity of KO, especially on day 7 and thereafter. The dissimilarity in functional diversity between the treatments was calculated by Bray-Curtis metric which demonstrated a distinct clustering of all treatments on day 0, and for SWD on days 3, 7, and 14, while the rest of the treatments overlapped (Supplementary Figure S4B). PERMANOVA analysis revealed that the treatment and incubation time were significant factors (*p* = 0.001) which explained 18% (*R*^2^ = 0.1814) and 21% (*R*^2^ = 0.2064), respectively, of the variability in KEGG orthologs.

To present the predicted functional abundance, we selected specific KO involved in aliphatic and aromatic hydrocarbon degradation pathways, as well as biosurfactant synthesis. For simplicity, we examined only the three treatments containing crude oil—BEWAF, CEWAF, and WAF. Samples from CEWAF treatment showed potential enrichment of genes involved in the degradation of medium-chain length alkanes, styrene, fluorobenzoate, polycyclic aromatic hydrocarbons (PAHs) (naphthalene, phenanthrene etc.), chlorocyclohexane, chlorobenzene, and xylene (Supplementary Figure S5). The relative abundance of genes which encode for the degradation of short-chain length (methane monooxygenase) and medium-chain length (alkane 1-monooxygenase and rubredoxin-NAD(+) reductase) alkanes were significantly increased in all three treatments on day 3, and in the CEWAF on days 3 and 7. The BEWAF treatment was enriched with genes involved in the degradation of chloroalkanes, benzoate, bisphenol, furfural, and flurobenzoate, while genes involved in the degradation of BTEX (benzene, toluene, ethylbenzene, xylene), dioxin, and nitrotoluene were predicted to be more abundant in the WAF treatment.

Genes for different biosurfactants biosynthesis were also predicted to understand the effect of the chemical dispersant and rhamnolipid on the ability of bacteria to produce biosurfactants. For example, genes involved in rhamnolipid synthesis, namely *rhl*A and *rhl*B (rhamnosyltransferases) were most abundant on days 0 and 3 in the BEWAF, CEWAF, and WAF treatments, although their abundance was not high (Supplementary Figure S5). Surfactin synthesis genes in the *srf*A operon (surfactin synthetase) were also predicted in these same treatments and time period, but with relatively higher abundance. Exopolysaccharide production protein ExoY was detected in all the treatments, but at lower abundance than for the rhamnolipid and surfactin genes on days 0 and 3, but it was comparatively higher on day 14 in the BEWAF treatment.

### Hydrocarbon biodegradation

**Fig. 6:**
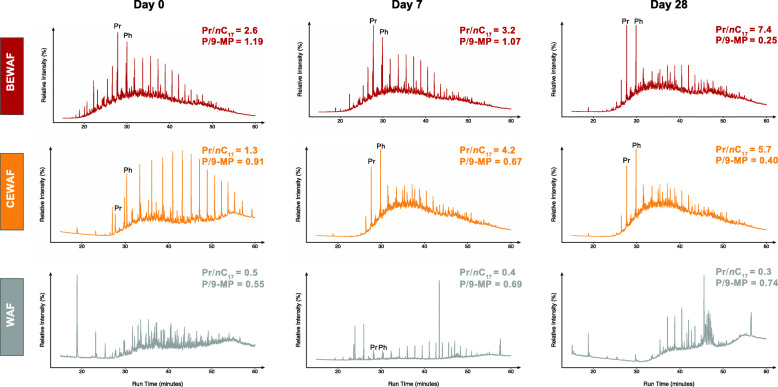
Representative flame-ionization chromatograms of the aliphatic hydrocarbon fraction of BEWAF (red), CEWAF (orange), and WAF (grey) through time of incubation (days 0, 7, and 28). Also shown are ratios of pristane versus heptadecane (Pr/*n*C17), which increases with increased biodegradation, and phenanthrene versus 9-methylphenanthrene (P/9-MP), which has an inverse relationship with biodegradation extent. Pristane (Pr) and phytane (Ph) are annotated for reference. Note ordinate axis is displayed in relative abundance

The GC-FID chromatograms for each oil-amended treatment were compared in order to assess the extent of degradation over the course of the incubation (Fig. 6). The peak areas for C_12_ to C_30_ *n*-alkanes, and two PAHs, phenanthrene and methylphenanthrene, were used to calculate ratios of specific hydrocarbons indicative of biodegradation (Supplementary Figure S6). The oil biodegradation in the rhamnolipid-amended treatments was relatively slow but insignificant (*p* > 0.05) in the first week of incubation, but it was significantly faster (*p* < 0.05) between day 14 and day 28 as indicated by pristane/*n*C_17_ ratio (Fig. 6). In contrast, oil biodegradation in the Finasol-amended treatment was initially rapid but slowed down over time, whereas biodegradation of alkanes in the WAF treatment was insignificant over time. PAH degradation was not significantly different across treatments or sampling times, though concentrations generally decreased over time in all treatments. The phenanthrene/9-methylphenanthrene ratio in the BEWAF treatment decreased the most by the end of the incubation compared to that in the CEWAF and WAF treatments (Fig. 6; Supplementary Figure S6) suggesting highest rates of PAH degradation in the BEWAF treatment.

## Discussion

The FSC is a cold subarctic environment (avg. *T* = 9.7 °C), so the in situ bacterial community was expectedly dominated by psychrophilic taxa, including known oil-degraders, such as *Oleispira*, *Colwellia*, and *Cycloclasticus*, that have been found globally to reside in cold sea surface [8, 34–36] and subsurface [37] waters, including in the FSC [38, 39]. The high abundance of oil-degrading bacteria in the region and their rapid (within 3 days) response to the crude oil, suggests they were primed from background exposure to hydrocarbons [40], possibly through permitted releases of produced water or from adjacent North Sea waterways, and frequent shipping and oil transportation activities in and around the FSC. Although no confirmed natural oil seeps are known in the FSC or nearby, evidence from satellite surveys showing oil slicks suggest subsurface oil seeps on the east and west of Scotland and offshore in the North Sea (Peter Browning-Stamp, pers. comm.). Natural seepage is known to prime a rich community of oil-degrading bacteria in the Gulf of Mexico [1] and the FSC appears to behave similarly.

*Colwellia* are commonly observed in cold surface and deep sea environments [36, 38, 41], and some members of the genus utilize a broad range of hydrocarbons, including short-chain alkanes [42], as well as PAHs, e.g., phenanthrene [43]. The metabolic versatility of *Colwellia* likely explained its early bloom in the WAF, BEWAF and CEWAF treatments but this may also result from their high in situ abundance in the FSC waters. In a recent study, *Colwellia* were implicated in dispersant-component degradation in treatments of deep-sea water from the Gulf of Mexico amended with the dispersant Corexit; their abundance increased from 1 to 43% after only 1 week [4]. Interestingly, in this study, *Colwellia* was the dominant organisms in the CEWAF treatment (by day 3), but in the dispersant-only treatment (SWD) the abundance of these organisms decreased markedly by day 3, and continued to decline thereafter subsequently becoming overprinted by members of the *Rhodobacteracaea* and *Vibrionaceae*; this pattern was similarly reported in another study using the synthetic dispersant Superdispersant-25 [38].

Members of the *Rhodobacteracaea* and *Vibrionaceae* may utilize oil-derived organic intermediates produced by hydrocarbon degraders [44, 45] or they may consume components of the dispersant itself, as previously shown in other studies using Corexit [4]. *Vibrio* became markedly enriched in only the SWD treatment, with highest levels reached by day 3. In contrast, *Vibrio* was less abundant in the CEWAF treatments, suggesting that these organisms might have a preference for the Finasol over the oil as a carbon and energy source. *Vibrio* are known for their quorum sensing ability and it is possible that their metabolic agility [46] allowed them to outcompete *Colwellia* in the SWD treatment. The observed bloom of the *Rhodobacteracaea*, mainly *Sedimentitalea*, *Pseudophaeobacter* and to a lesser extent *Sulfitobacter*, may have contributed to oil degradation as members of these genera have hydrocarbon degrading capabilities [39, 45, 47].

A previous study [4] employed the dispersant Corexit 9500, whose composition (18% DOSS, 4.4% Span 80, 18% Tween 80, and 4.6% Tween 85) is distinct from Finasol (15–25% DOSS, 15–23% non-ionic carboxylic acids and alcohols). The presence of other surfactants in Finasol may have promoted a sustained response by *Vibrio*, especially in the dispersant only (SWD) treatment. A previous study [38] found that *Vibrio* responded strongly to Corexit amendment, mirroring observations of increased *Vibrio* abundance in impacted Gulf of Mexico surface water samples collected during the active discharge phase of the incident [48]. Techtmann et al. [49] suggested that *Vibrio* metabolized metabolic by-products of oil degradation by other microorganisms. This could not be the mechanism in this case; however, since there is no oil in the SWD treatment, it is possible that *Vibrio* were metabolizing the carboxylic acids and alcohols in the dispersants. Similar compounds are known intermediates of oil biodegradation [50].

*Alcanivorax* was only observed in oil-amended treatments; it was undetectable in the controls (SW, SWD and SWBS). *Alcanivorax* is recognized for its almost exclusive preference for aliphatic hydrocarbons and for commonly blooming soon after oil is introduced to an environment [14, 35, 38]. However, *Alcanivorax* increased later (days 14 and 28) in the WAF, BEWAF, and CEWAF treatments. This was unexpected and may be because they were outcompeted by more resilient earlier bloomers (esp. *Colwellia*, *Oleispira*, *Sedimentitalea*, and uncultured *Micavibrionaceae*), which is reminiscent to the non-enrichment of *Alcanivorax* during the DWH oil spill. 16S rRNA gene sequences for this genus were undetected in water column metagenomic libraries from the Gulf of Mexico during the spill’s most active phase [41, 43]. *Alcanivorax* in surface waters of the FSC may have access to a greater variety of hydrocarbons when grown on dispersed oil (for example in the CEWAF treatment), as observed elsewhere [51], or the *in situ* cold temperatures (~ 10 °C) and/or nutrient limitation in the FSC may have delayed their response.

One of the early bloomers, *Cycloclasticus*, a microbe known for its appetite for PAHs [14, 43], appeared by day 7 in the WAF treatment predominantly, where it increased in abundance until day 28. *Cycloclasticus* also appeared in BEWAF treatment towards the end of the incubation. *Cycloclasticus* was not observed in the Finasol-amended treatments, which was surprising since previous studies showed *Cycloclasticus* domination in the microbial communities in CEWAF amendments of northeast Atlantic seawater [8, 38] and in natural deepwater oil plumes during the early phase of the DWH spill [45, 47]. However, all of these studies used different types of a synthetic dispersant. Comparing the dynamics of *Cycloclasticus* across the Finasol- and biosurfactant-amended treatments, it was clear that Finasol negatively impacted this taxon. This inhibition of *Cycloclasticus* has profound implications for oil biodegradation since it is a known key player in the biodegradation of aromatic hydrocarbons [14]. The absence of *Cycloclasticus* might have translated to reduced biodegradation rates for the aromatic fraction in CEWAF treatments.

We observed relatively high abundance of an uncultured member of the family *Micavibrionaceae* in the in situ FSC microbial community, initially across all treatments, in a later bloom in the BEWAF and SWBS treatments, and to a lesser extent in the WAF (14%) by days 14 and 28. The order *Micavibrionales* is assigned to a group of obligate predatory bacteria, the *Bdellovibrio*, and like organisms (BALOs) [52]. Although species from this order were first described in 1982 [53], not much is known about them or their role in natural ecosystems. A recent study from Lake Geneva [54] demonstrated that *Micavibrionaceae* vary throughout the year, with higher numbers in the spring, likely linked to phytoplankton dynamics since they are possible prey for BALOs. Their abundance here may relate to the time of year as our sampling coincided with a phytoplankton bloom. The dynamics of *Micavibrionaceae* may also result from their preying on blooming oil-degrading bacteria, as their abundance increase coincided with decreasing numbers of *Colwellia* and *Oleispira* in the WAF, BEWAF, and SWBS treatment by days 14 and 28. Such top-down grazing control of oil degrading organisms is not usually considered when assessing their dynamics, but it is clear that this may be quite important in influencing the composition and biodegradation capability of oil-degrading microbial communities.

As expected, we found that environmental filtering strongly determined the local community composition of surface seawater communities when enriched with crude oil in combination with either Finasol or rhamnolipid. In other words, the crude oil, dispersant, and/or rhamnolipid limit community membership whereby closely related (e.g., belonging to the same family) and ecologically similar (e.g., hydrocarbon-degrading) taxa are more likely to coexist than expected if random ecological processes (drift) assembled the composition. The environmental filtering, however, was measured to be the strongest in the Finasol-amended communities, which displayed the lowest diversity during the incubation period and were characterized by the quicker and stronger response of members of only two families, *Rhodobacteracaea* and *Vibrionaceae*, which contain members of known opportunistic oil degraders [14]. The regression analysis we employed in our study confirmed that oil dispersed by Finasol had a significant negative influence on community structure correlated with decreased species richness and increased local contribution to beta diversity.

Our analysis of robustness revealed interesting differences between communities from the varying treatments. Although robustness to taxonomic perturbations has not been directly compared between treatments experimentally, there may be some evidence that supports chemically-dispersed oil communities (CEWAF) being more susceptible to disturbed function than non-treated or rhamnolipid-dispersed oil communities. There were noticeable alterations to taxonomic composition in the CEWAF and SWD treatments compared to the rest of the treatments which can be associated with changes in the community functional capacities [24, 55]. Lower taxa-function robustness may be selected to enable flexible functional response to a changing environment, as for example by selecting for generalist and opportunistic species that can modulate the community functional profile in a desired direction. Indeed, the taxonomic composition in the CEWAF and SWD treatment shifted from being dominated by obligate hydrocarbon degraders in the early stages of the incubation to generalist and opportunistic taxa by the end of the incubation.

Whilst metabolic potential from 16S rRNA studies are often discounted as mere predictions, the newer version of PICRUSt2 has a comprehensive reference database (> 20,000 genomes covered as opposed to its predecessor which only had ~ 2000), and a very high correlation with matched metagenomics datasets (~ 0.9) [25]. The majority of the ASVs in our datasets were represented in the PICRUSt2 reference database; therefore, this analysis has a very strong utility to give mechanistic understanding of the functional profiling in our microcosm communities. Results of PICRUSt2 analysis suggested that structurally dissimilar communities could embody similar functions, supportive of the functional redundancy [56] to microbial communities. The abundance of some biosurfactant-producing genes was investigated to gain insights into the effect of Finasol and rhamnolipid on the ability of bacteria to synthesize biosurfactants and degrade hydrocarbons. Although genes involved in the biosurfactant biosynthesis were predicted by PICRUSt2, the search for such genes was limited to only a few well-known genes (e.g., rhamnosyltransferases and surfactin synthetase) due to the current knowledge bottleneck in regards with microbial biosurfactant biosynthesis pathways [57]. According to previous reports, adding rhamnolipids to bacterial cultures enhances the biodegradation of >*n*C15 alkanes [16, 17, 58]. Typically, rhamnolipid production is associated with pathogenic *Pseudomonas* spp. Recent reports have shown rhamnolipid production by non-pathogenic species of *Pseudomonas* isolated from the marine environment [59, 60], as well as form other bacterial species including *Burkholderia thailandensis* E264 [61], *Acinetobacter calcoaceticus* and *Enterobacter asburiae* [62], and more recently form *Marinobacter* [63]. In our study, the relative abundance of *Pseudomonas* was only notable in the Finasol-amended microcosms. However, rhamnolipid genes were predicted in all oil-amended treatments (WAF, CEWAF, and BEWAF), though in low abundance, indicating that rhamnolipid synthesis was not limited to the presence of *Pseudomonas* and that other members of the community were also involved. The extent to which the biosurfactant synthesis was inhibited or stimulated by the chemical dispersant and rhamnolipid remains to be determined. Nevertheless, a recent proteome-level investigation revealed that under specific environmental conditions (e.g., starvation), exposure to chemical dispersant (Corexit) can induce changes in cellular processes including EPS secretion and aggregate formation [64, 65], which could explain the low abundance of predicted biosurfactant genes in the late stages of the microcosm incubations in our study.

Not surprisingly, hydrocarbon degradation pathways were predicted in the three oil-amended treatments (WAF, BEWAF, and CEWAF). Although it demands further confirmation by shotgun sequencing, genes involved in medium length alkanes and PAHs degradation were predicted to be more enriched in the chemically dispersed oil treatment (CEWAF) compared to the oil-only (WAF) and biosurfactant-dispersed oil treatments (BEWAF). However, the GC-FID analysis revealed that PAH degradation appeared to be limited in the CEWAF treatment, which is in agreement with previously reported results that used chemically enhanced WAF microcosms design [4]. As mentioned above, by day 28 the microbial community in the CEWAF treatment was characterized by the absence of the obligate PAH degrader *Cycloclasticus* (and other obligate degraders) and the dominance of family *Rhodobacteriaceae* (~ 55% of the total community). It is plausible to assume that the predicted enrichment of PAH genes in the CEWAF treatment was related to the dominance of *Rhodobacteriaceae*. The role of *Rhodobacteriaceae* in PAH degradation has been documented in a study which employed 16S rRNA-based microarray (PhyloChip) to successfully replicate the enrichment and succession of the predominant oil-degrading bacterial taxa observed during the DWH event [45]. It is possible that *Rhodobacteriaceae* in our study were not as effective in degrading PAHs as evident by the slower degradation revealed by the GC-FID analysis. The relatively low seawater temperature (~ 10 °C) and/or in combination with nutrient exhaustion could also be contributing to the slower degradation of PAHs [66]. In contrast, by day 28 the relative abundance of *Cycloclasticus* was high in the WAF and BEWAF treatments which appeared to confer the higher PAH degradation in these treatments.

While chemical dispersants (inlc. Finasol) are highly effective in dispersing hydrocarbons and stimulate biodegradation, they tend to select for generalist taxa and against some of the most effective specialist hydrocarbonoclastic bacteria (e.g., *Cycloclasticus*). Our study has provided evidence that rhamnolipid biosurfactant is able to enhance hydrocarbon degradation as effectively as the commercial dispersant Finasol, and without suppressing specialist hydrocarbonoclastic bacteria and, thus, offering what appears to be a slight advantage over Finasol. Rhamnolipids have been shown to stimulate the growth of hydrocarbon degraders in microcosms, resulting in the utilization of up to 50% of the saturated hydrocarbons present [16]. Although rhamnolipids are the most studied and widely commercialized biosurfactants on the market, their large-scale production remains a considerable challenge due to low yields and high production costs [67]. In order to fully or partially (e.g., as part of the dispersant’s composition) replace chemical dispersants, more research and investment is needed to develop highly efficient downstream processes that can maximize biosurfactant yields, improve or alter physico-chemical properties as desired, and overall increase their economic viability.

## Conclusions

Our results demonstrate that there was a differential distribution of bacterial communities in seawater amended with oil, oil with synthetic dispersant, and oil with rhamnolipid over time. However, a number of common taxonomic genera that are known obligate and generalist hydrocarbon degraders, were observed in abundance in all treatments (including the in situ non-treated FSC community), especially in the early days of the incubation period. Over time, the microbial succession patterns dramatically changed and triggered by the presence of Finasol. A comprehensive set of analyses revealed that Finasol played a major role influencing microbial dynamics by negatively impacting diversity, weakened the taxa-functional robustness, and caused a stronger environmental filtering, more so than oil-only and rhamnolipid-amended oil treatments. Nevertheless, Finasol stimulated faster *n*-alkane degradation, but it suppressed biodegradation of the aromatic fraction which corroborates with the suppression of obligate aromatic hydrocarbon degraders, such as *Cycloclasticus*. The presence of rhamnolipid, on the other hand, supported higher diversity of obligate hydrocarbon degrading taxa, such as *Oleispira*, *Alcanivorax*, and *Cycloclasticus*. However, while our study was performed in laboratory-scale microcosms, microbial ecology is expected to be relatively more complex in full-scale marine oil spills and, therefore, our results should serve as guidance for the decision-making process for oil spill response because of the great variation of field scenarios and environmental conditions.

## Supplementary Information

**Additional file 1: Supplementary methods.** Supplementary Methods and Materials used to produce the main results in this study.

**Additional file 2: Supplementary File 1.** Top 25 taxa relative abundance. Relative abundance of top 25 taxa in all treatments (including replicates) over time.

**Additional file 3: Supplementary Figure S1.** Local contribution to beta diversity (LCBD) for Bray-Curits, Unweighted and Weighted UniFrac distance matrices. Colours represent incubation time: red – in situ FSC at time of sampling, olive green – day 0, green – day 3, blue – day 7, pink – day 14 and brown – day 28. FSC is the *in-situ* baseline microbial community, WAF - seawater and oil only, BEWAF – seawater, crude oil and biosurfactant, CEWAF – *seawater,* crude oil and synthetic dispersant, SW - seawater only, SWBS - seawater and biosurfactant, and SWD – seawater and synthetic dispersant. Statistically different treatments (pair-wise ANOVA) are annotated with bracket and the level of significance is shown above the bracket with a star sign: * is *p* < 0.05, ** is *p* < 0.01, and *** is *p* < 0.001.

**Additional file 4: Supplementary Figure S2.** Summary of significant predictive parameters (i.e., treatments and incubation days) determined by regression analysis based on unsupervised machine learning for two alpha diversity measures (Richness and Shannon index), NTI, NRI, and Local contribution to beta diversity (LCBD). The parameters shown here are from the optimal model for each metric, where blue represent negatively and red – positively influencing variables, respectively. FSC is the *in-situ* baseline microbial community, WAF - seawater and oil only, BEWAF – seawater, crude oil and biosurfactant, CEWAF – seawater*,* crude oil and synthetic dispersant, SW - seawater only, SWBS - seawater and biosurfactant, and SWD – seawater and synthetic dispersant.

**Additional file 5: Supplementary File 2.** Differential abundant taxa. DESeq2 results for the differential expressed bacterial taxa between treatments BEWAF (seawater, crude oil and biosurfactant), WAF (seawater and crude oil only) and CEWAF (seawater, crude oil and synthetic dispersant).

**Additional file 6: Supplementary Figure S3.** Differential heat trees showing the key (significant) differential taxa (DESeq2; Wilcoxon *p*-value test adjusted with multiple comparison) in seawater-only control treatment (SW). The top 3 subsets with the highest correlation with the full ASV table considering Bray-Curtis distance (PERMANOVA) are listed for each treatment. The grey tree is the taxonomy key for the smaller unlabelled coloured trees. The colour of each taxon represents the log-10 ratio of median proportions of reads observed in each treatment. The size of tree nodes shows the number of ASVs (here labelled as OTUs) present in each sample.

**Additional file 7: Supplementary Figure S4.** (A) Predicted functional alpha diversity of microbial pathways (expressed as number of KEGG orthologs). Statistically different treatments (pair-wise ANOVA) are connected by bracket and the level of significance is shown with: *(*p* < 0.05), ** (*p* < 0.01), or *** (*p* < 0.001). (B) Principal coordinate analysis (PCoA) on beta diversity measured with Bray-Curtis dissimilarity distance matrix. In both (A) and (B) treatments are represented by shape (shown on graph) and incubation time by colour: red – baseline microbial community at time of seawater sampling, olive green – day 0, green – day 3, blue – day 7, pink – day 14, and brown – day 28. FSC is the *in-situ* baseline microbial community, WAF - seawater and oil only, BEWAF – seawater, crude oil and biosurfactant, CEWAF – seawater*,* crude oil and synthetic dispersant, SW - seawater only, SWBS - seawater and biosurfactant, and SWD – seawater and synthetic dispersant.

**Additional file 8: Supplementary Figure S5.** Heatmap showing the scaled log abundance (color key on top left) of aliphatic and aromatic degradation, and biosurfactant synthesis pathways. Pathways are shown along the y-axis and BEWAF (seawater, crude oil, and biosurfactant), CEWAF (seawater, crude oil, and synthetic dispersant), and WAF (seawater and crude oil only) samples along the x-axis. The two color-coded bars on top of the heatmap indicate their treatments and incubation days status. Hierarchical clustering of the samples (top) is based on the correlation between samples’ predicted gene expression.

**Additional file 9: Supplementary Figure S6.** Differences in aliphatic and polycyclic aromatic hydrocarbon biomarker ratios of three different treatments amended with crude oil: BEWAF (seawater, crude oil, and biosurfactant), CEWAF (seawater, crude oil, and synthetic dispersant), and WAF (seawater and crude oil only), over time in days (grey boxes): nC17/pristane (nC17/pri), nC18/phytane (nC18/phy), Phenanthrene/9-methylphenanthrene (P/9MP), (3+2)-methylphenanthrene/(9+1)-mehylphenanthrene (3+2MP/9+1MP), and 3-methylphenanthrene/9-methylphenanthrene (3MP/9MP). Values are the mean of three independent replicates (except for BEWAF day 0 (two replicates) and CEWAF day 28 (one replicate)) +/- standard deviation.

## Data Availability

The raw sequences files supporting the results of this article are available in the NCBI Sequence Read Archive under accession number PRJNA636672.
