# Supplementary material for: Response and oil degradation activities of a northeast Atlantic bacterial community to biogenic and synthetic surfactants
Source: Microbiome. 2021 Sep 21;9:191. doi: 10.1186/s40168-021-01143-5 (PMC8456599; doi:10.1186/s40168-021-01143-5)
Supplement: Supplementary file 2 — Additional file 1: Supplementary methods. Supplementary Methods and Materials used to produce the main results in this study. [file 40168_2021_1143_MOESM2_ESM.docx]

**Supplementary Methods**

**Field sample collection in the Faroe-Shetland Channel**

During a research cruise on MRV *Scotia* in May of 2018, surface seawater (3m depth) was collected from the Faroe-Shetland Channel (FSC), a subarctic region of the northeast Atlantic. The seawater temperature at the time of collection was measured at 9.7 °C and the salinity 35.28‰. This sampling site lies on the Fair Isle-Munken line (1) near the Foinaven oil field development area, approximately 3 and 9.3 nautical miles respectively from the Petrojarl Foinaven and Glen Lion production facilities. Collection of seawater samples was performed using 10 L Niskin water bottles mounted on a CTD (conductivity, temperature, depth) carousel. Immediately after recovery, some of the collected seawater was used to rinse, at least three times, two Nalgene carboys (10L each; acid-washed, acetone-rinsed and dried) prior to filling, and immediately stored at 10 °C onboard the vessel for transport back to the laboratory for preparation of water-accommodated fractions (WAFs) and for the crude oil and biosurfactant/dispersant enrichment experiments, as described below. Sub-samples were collected for DNA extraction and quantification of the *in-situ* bacterial communities and to account for any changes before initiation of experiments (described below). For this, 3 litres of the sampled water were filtered through polycarbonate membrane filters (1L per filter; 0.22μm pore size) and the filters stored onboard at -20 °C until return to the laboratory when they were transferred to -80 °C for subsequent analysis.

**DNA extraction and barcoded amplicon 16S rRNA gene sequencing**

Filters with collected biomass from each treatment (incl. replicates) were each transferred into sterile 1.5 ml Eppendorf tubes and submerged in liquid nitrogen until completely frozen. The frozen filters were carefully crushed into fine particles using sterile pipette tips. DNA was extracted according to the method of a previous study (2). Sterile deionised water samples were included to act as negative controls to qualify the sterility of the method and of the reagents used. DNA extracts were resuspended in 20 μl of 1 mM TE buffer and confirmed by gel electrophoresis prior to storage at -20 °C for Illumina barcoded-amplicon sequencing.

A two-step amplification procedure was used to minimize the heteroduplex formation in mixed-template reactions (3). The 16S rRNA gene was first amplified with universal bacterial primers 8F (5`-AGAGTTTGATCCTGGCTCAG-3`) (4) and 1492R (5`-TACGGYTACCTTGTTACGACT-3`) (5) in triplicate 25 μl reactions each containing 2.5 U MyTaq™ Red DNA Polymerase (Bioline Reagents Ltd.), 5 μl 5x MyTaq Red Reaction buffer (containing 5 mM dNTPs and 15 mM MgCl_2_) (Bioline Reagents Ltd.), 17.5 μl molecular grade water, forward and reverse primer at final concentration of 0.2 μM each, and 1 μl of target DNA. Amplification was carried out on a thermocycler using the following conditions: initial denaturation at 96 °C for 5 min, followed by 32 cycles of 96 °C for 30 sec, 54 °C for 30 sec and 72 °C for 30 sec, and a final extension at 72 °C for 10 min. Purified samples were quantitated with Nanodrop and stored at -20 °C for the second step of barcoded amplification.

The second step involved the targeted barcoded amplification of the V4 hypervariable region of the 16S rRNA gene from the first-step PCR. For each sample, this was performed using duplicate 25 μl reactions. Each reaction consisted of the same reagents as in the first PCR step but this time barcoded 515F (5′-GTGYCAGCMGCCGCGGTAA-3′) (6) and 806R (5′-GGACTACNVGGGTWTCTAAT-3′) (7) primers were added to the PCR mixture instead of 8F and 1492R primers. Both primers contained Illumina MiSeq adapters added to the 5′ ends and unique Golay barcodes added to the 515F primer. Barcoded amplification was carried out as per Earth Microbiome project’s thermocycler conditions (8).

**Statistical analyses**

***Alpha and beta diversity***

Alpha diversity indices Shannon and Species Richness were calculated on the rarefied-to minimum-reads ASVs to investigate within-community diversity of each treatment through time. Variance between treatments at different incubation times for each alpha diversity index were compared by performing ANOVA. Community composition variation between treatments and incubation time (Beta diversity) were assessed using the adonis function in vegan package (9) to perform pairwise Permutational Multivariate Analysis of Variance (PERMANOVA). Principal Coordinate Analysis plots (PCoA) were used to visualise dissimilarities based on Bray-Curtis similarity matrix and Weighted UniFrac distance matrices. Significance of results was based on high F values and level of significance based on low p-values (<0.05) for a comparison to be considered a significantly different.

***Homogeneity variances***

To understand multivariate homogeneity of groups, variances between multiple conditions (combinations of treatments over time), vegan’s betadisper function was used, in which the distances between objects and group centroids are handled by using a reduced order representation based on distance metrics (Bray-Curtis or Weighted UniFrac) in principal coordinates space and afterwards performing ANOVA on differences of each sample from the mean of the group they belong to. Vegan’s adonis function was used for multivariate analysis of variance (PERMANOVA) among sources of variation (treatment and sampling time) using distance matrices (Bray-Curtis /Weighted Unifrac). To determine which ASVs were significantly different between multiple conditions (treatments/days), DESeq2 package (10) was used. This function uses negative binomial GLM-fitting and wald statistics prior to applying Bayesian shrinkage.

***Local contribution to beta diversity (LCBD)***

To show how each sample was markedly different in terms of beta diversity, we have performed LCBD analysis using adespatial package (11). The procedure calculates the total beta diversity considering all the samples, and then allocates the proportions based on how different the microbial community structure of a single sample is from the average (with higher LCBD values representing outliers) beta diversity, and also provides a mean to show when the community structure has stabilised in a temporal setting (12).

***Ecological drivers***

To better understand how the environment influences the microbial community structure, we quantified the nearest-taxon-index (NTI; local phylogenetic clustering) and nearest-relative-index (NRI; global phylogenetic clustering) by adopting the approach of Stegen et al. (13). NRI and NTI were calculated using ses.mpd() and ses.mntd() functions, respectively, from the picante package (14).

***Subset regression analysis***

We performed subset regression of different metrics of microbiome (alpha and beta diversity) by testing all possible combination of the predictor variables (in our case, categorical variables), and then selecting the best model according to some statistical criteria, with recommendations given by Kassambra et al. (15) with their code available online [here](http://www.sthda.com/english/articles/37-model-selection-essentials-in-r/155-best-subsets-regression-essentials-in-r/). The R function regsubsets() from leaps package (16) was used to identify different best models of different sizes, by specifying the option nvmax, set to the maximum number of predictors to incorporate in the model. Having obtained the best possible subsets, the k-fold cross-validation consisting of first dividing the data into k subsets. Each subset (10%) served successively as test data set and the remaining subset (90%) as training data. The average cross-validation error was then computed as the model prediction error. This was computed using a custom function utilising R’s train function from the caret package (17) and tab_model function from sjPlot package (18).

***Predicted taxa-functional relationship***

To categorise the predicted functional diversity, we used KEGG (Kyoto Encyclopedia of Genes and Genomes) pathway mapping tool and MetaCyc databases (19). KEGG orthologs (KO) involved in aliphatic and aromatic hydrocarbon degradation were manually filtered along with biosurfactant-associated enzymes for each treatment. We specifically looked at genes for rhamnolipid (glycolipid) and surfactin (lipopeptide) synthesis as they are the most studied and these metabolic pathways are well known. Because our study focused on a marine microbial community, we also selected genes for exopolymer production which are more characteristic in marine bacteria, for example *Alcanivorax* sp. and *Halomonas* sp., and also genes encoding the *rhl* quorum sensing system in rhamnolipid-producing strains that regulates the production of rhamnolipid biosurfactants.

***Predicted taxa-function robustness***

The taxa-function robustness measure of the microbial communities was determined by following the method of Eng and Borenstein (20). Briefly, the raw 16s rRNA sequences were used in QIIME2 to generate functional profiles based on the KO groups assigned to the Greengenes database (gg_13_5) (21). Each of the 90 samples (across 7 treatments) was perturbed 100 times to calculate a two-dimensional taxonomic shift versus functional shift profile. For the taxonomic shift, the phylogeny-aware weighted UniFrac dissimilarity metric was calculated between the original sample and its perturbations, and for the functional shift, cosine dissimilarity was used between the functional profiles of the original community and its perturbations. Next, the relationship between taxonomic perturbation magnitude and functional profile shift are fitted using the linear regression model on natural log-transformed data: $\ln\left( ƒ \right)= -a +b \ln\left( t \right)$ to estimate two coefficients: attenuation *a***,** and buffering *b*. Attenuation is described as the expected rate at which increases in the taxonomic perturbation magnitude are expected to increase functional shifts, and buffering specifies exactly how large a perturbation must be before a functional profile shift becomes apparent. The attenuation and buffering coefficients serve as proxy (robustness factors) to summarise the property of a sample, and then were calculated for all samples in the dataset (20). As the attenuation describes the slope of the response curve, it means that larger taxonomic perturbations should yield larger functional shifts.

**Hydrocarbon degradation analysis by GC-FID/MS**

Replicate bottles (300-ml volume per bottle) were sacrificed for hydrocarbon extraction at three time points – on days 0, 7 and 28. Total hydrocarbons were extracted via solvent extraction using dichloromethane (DCM; HPLC grade, ThermoFisher, UK) at an oil/water mix to DCM ratio of 1:1. The DCM fraction was removed into acid-washed and dried pre-weighted 500-ml round-bottom flasks and the aqueous phase re-extracted with DCM twice more. Combined DCM extracts were then rotary evaporated to ~2 ml at 40°C and transferred to amber glass vials and stored at -20 °C for subsequent analysis by GC-FID/MS.

A known aliquot corresponding to ca. 10 mg of total hydrocarbon content was taken from each sample and transferred to a 10 ml vial and dried down using a gentle stream of nitrogen gas. Sample residues were dissolved in a small volume of hexane (~200 µl). A methodological blank (no crude oil) was used to ensure no contamination from any residual hydrocarbons during the analysis, and a sample of the original Schiehallion crude oil was analysed as a reference. Total hydrocarbon extracts were separated into two complementary polarity-defined fractions (aliphatic and aromatic) by using so-called *flash* open-column chromatography over 0.5 g silica gel (0.060 – 0.200mm, 60A) and 0.5 g alumina (aluminium oxide, 50-200μm, 60A) sorbents. Sorbents were pre-extracted with DCM to minimize organic contaminants and then activated at 120 °C prior to use. Sorbents were introduced to open columns as slurries in hexane. Each column was packed with half the alumina, topped with silica gel, and then alumina again. The column was flushed with at least two bed-volumes of hexane (~4 ml) before the sample, which was dissolved in hexane, was applied to the top of the column. The aliphatic fraction of the total petroleum hydrocarbons (TPH) was eluted with 4 ml of hexane into an organics-free 2 ml glass vial. The aromatic fraction of the TPH was eluted with 4 ml DCM into a separate vial. Elution solvents were dried-down under nitrogen, re-dissolved in hexane (aliphatic fraction) and DCM (aromatic fraction) and transferred to organics-free 300 µl GC/MS borosilicate vial inserts. The samples were dried as before with nitrogen gas. The TPH fractions were then analysed by injecting 1 µl of the hydrocarbon fraction diluted in hexane in the autosampler of a Thermo Trace 1310 GC coupled with Thermo ISQ LT MS and fitted with a splitless constant temperature injector (300 °C), a flame ionisation detector (FID) at 310 °C, and an HP-5MS capillary column (30m x 0.25mm x 0.2µm; Agilent). The column programme was set at 50 °C for 2 min and 5 °C/min to 310 °C for 21 min to a total run time of 75 minutes. Chromatographic data were acquired and processed through Chromeleon software (v. 7.2.8; Thermo Fisher). Peak areas of individual C_12_ to C_30_ *n*-alkanes and the isoprenoids pristane and phytane were calculated. The aromatic hydrocarbons were analysed by GC-MS in full scan mode (50-600 amu at 4 min).

Peak areas of aliphatic and aromatic hydrocarbon species/groups that were biodegraded after 7 or 28 days were calculated by subtracting the respective hydrocarbon concentrations measured in the control from those of the treatment incubations. Additionally, ratios of *n*-alkanes to acyclic isoprenoid hydrocarbons (*n*C_17_/pristane and *n*C_18_/phytane) were used as conventional indicators of biological degradation, due to the recalcitrance imparted by the branched structure of the isoprenoid biomarkers (22). Similarly, for the aromatic hydrocarbons, four ratios indicative of biodegradation were determined (phenanthrene/9-methylphenanthrene, 3+2-methylphenanthrene/9+1-methylphenanthrene, 3-methylphenanthrene/9-methylphenanthrene) (22). Two-way ANOVA and *post-hoc* Tukey tests were performed to test for significant differences in the degradation of the hydrocarbons analysed between the treatments.

**References**

1. Turrell WR, Slesser G, Adams RD, Payne R, Gillibrand PA. Decadal variability in the composition of Faroe Shetland Channel bottom water. Deep-Sea Research Part I: Oceanographic Research Papers. 1999;46(1):1–25.

2. Tillett D, Neilan BA. Xanthogenate nucleic acid isolation from cultured and environmental cyanobacteria. Journal of Phycology. 2000;36(1):251–8.

3. Berry D, Ben Mahfoudh K, Wagner M, Loy A. Barcoded primers used in multiplex amplicon pyrosequencing bias amplification. Applied and environmental microbiology. 2011 Nov 1;77(21):7846–9.

4. Galkiewicz JP, Kellogg CA. Cross-Kingdom Amplification Using Bacteria-Specific Primers: Complications for Studies of Coral Microbial Ecology. APPLIED AND ENVIRONMENTAL MICROBIOLOGY. 2008;74(24):7828–31.

5. Chirino B, Strahsburger E, Agulló L, González M, Seeger M. Genomic and Functional Analyses of the 2-Aminophenol Catabolic Pathway and Partial Conversion of Its Substrate into Picolinic Acid in Burkholderia xenovorans LB400. PLoS ONE. 2013;8(10).

6. Parada AE, Needham DM, Fuhrman JA. Every base matters: Assessing small subunit rRNA primers for marine microbiomes with mock communities, time series and global field samples. Environmental Microbiology. 2016;18(5):1403–14.

7. Apprill A, Mcnally S, Parsons R, Weber L. Minor revision to V4 region SSU rRNA 806R gene primer greatly increases detection of SAR11 bacterioplankton. Aquatic Microbial Ecology. 2015;75(2):129–37.

8. Caporaso JG, Lauber CL, Walters WA, Berg-Lyons D, Lozupone CA, Turnbaugh PJ, et al. Global patterns of 16S rRNA diversity at a depth of millions of sequences per sample. PNAS. 2011;108(Supplement_1):4516–22.

9. Oksanen AJ, Blanchet FG, Friendly M, Kindt R, Legendre P, Mcglinn D, et al. Community ecology package ‘ vegan ’. R package version 2.5-6. 2019;

10. Love MI, Huber W, Anders S. Moderated estimation of fold change and dispersion for RNA-seq data with DESeq2. Genome Biology. 2014;15(12):1–21.

11. Dray AS, Blanchet G, Borcard D, Guenard G, Jombart T, Larocque G, et al. Package ‘adespatial’. R package version 0.0-8. CRAN; 2017.

12. Ijaz UZ, Sivaloganathan L, McKenna A, Richmond A, Kelly C, Linton M, et al. Comprehensive longitudinal microbiome analysis of the chicken cecum reveals a shift from competitive to environmental drivers and a window of opportunity for Campylobacter. Frontiers in Microbiology. 2018;9(OCT):1–14.

13. Stegen JC, Lin X, Konopka AE, Fredrickson JK. Stochastic and deterministic assembly processes in subsurface microbial communities. ISME Journal. 2012;6(9):1653–64.

14. Kembel SW, Cowan PD, Helmus MR, Cornwell WK, Morlon H, Ackerly DD, et al. Picante: R tools for integrating phylogenies and ecology. Bioinformatics. 2010;26(11):1463–4.

15. Kassambara A. Machine Learning Essentials: Practical Guide in R. STHDA; 2018. 211 p.

16. Lumley T. Leaps: Regression subset selection. R package version 3.1. 2020;

17. Kuhn M. caret Package. Journal Of Statistical Software. 2008;28(5):1–26.

18. Lüdecke D. sjPlot - Data visualization for statistics in social science. Zenodo; 2014. p. 2016.

19. Caspi R, Billington R, Keseler IM, Kothari A, Krummenacker M, Midford PE, et al. The MetaCyc database of metabolic pathways and enzymes-a 2019 update. Nucleic Acids Research. 2019;48:445–53.

20. Eng A, Borenstein E. Taxa-function robustness in microbial communities. Microbiome. 2018;6(45):19.

21. DeSantis TZ, Hugenholtz P, Larsen N, Rojas M, Brodie EL, Keller K, et al. Greengenes, a chimera-checked 16S rRNA gene database and workbench compatible with ARB. Applied and environmental microbiology. 2006 Jul 1;72(7):5069–72.

22. Dawson KS, Schaperdoth I, Freeman KH, Macalady JL. Anaerobic biodegradation of the isoprenoid biomarkers pristane and phytane. Organic Geochemistry. 2013 Dec 1;65:118–26.

23. Chong J, Liu P, Zhou G, Xia J. Using MicrobiomeAnalyst for comprehensive statistical, functional, and meta-analysis of microbiome data. Nature Protocols. 2020 Mar 15;15(3):799–821.
