# Supplementary figures and images for: Response and oil degradation activities of a northeast Atlantic bacterial community to biogenic and synthetic surfactants
Source: Microbiome. 2021 Sep 21;9:191. doi: 10.1186/s40168-021-01143-5 (PMC8456599; doi:10.1186/s40168-021-01143-5)

Bray-Curtis

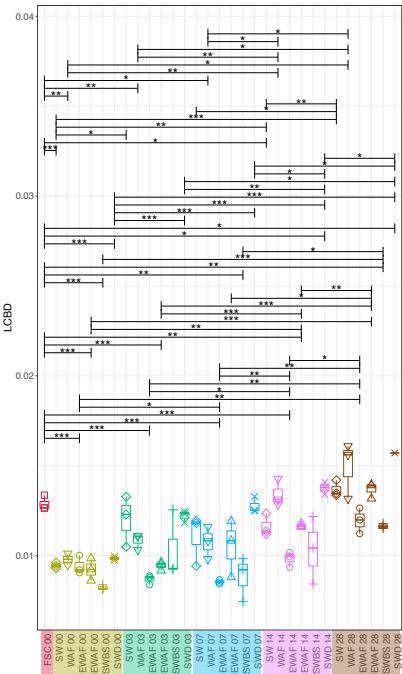

Unweighted UniFrac

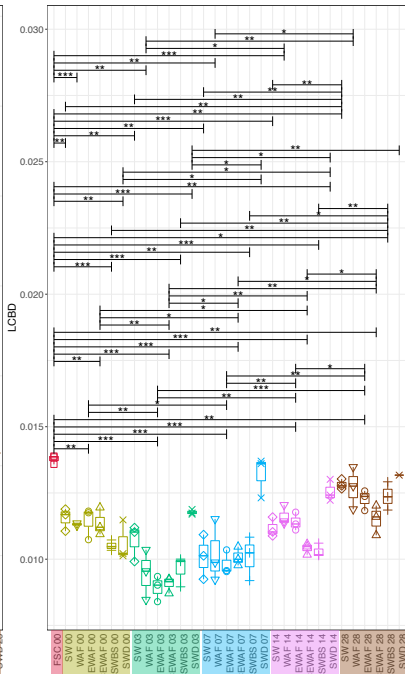

Weighted UniFrac

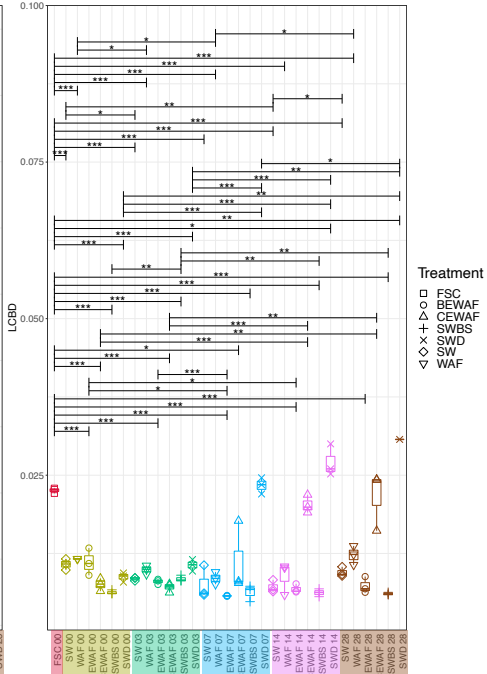

Treatment

□ FSC  
 ○ BEWAF  
 △ CEWAF  
 + SWBS  
 × SWD  
 ◇ WAF

Supplement: Supplementary file 4 — Additional file 3: Supplementary Figure S1. Local contribution to beta diversity (LCBD) for Bray-Curits, Unweighted and Weighted UniFrac distance matrices. Colours represent incubation time: red – in situ FSC at time of sampling, olive green – day 0, green – day 3, blue – day 7, pink – day 14 and brown – day 28. FSC is the in-situ baseline microbial community, WAF - seawater and oil only, BEWAF – seawater, crude oil and biosurfactant, CEWAF – seawater, crude oil and synthetic dispersant, SW - seawater only, SWBS - seawater and biosurfactant, and SWD – seawater and synthetic dispersant. Statistically different treatments (pair-wise ANOVA) are annotated with bracket and the level of significance is shown above the bracket with a star sign: * is p < 0.05, ** is p < 0.01, and *** is p < 0.001. [file 40168_2021_1143_MOESM4_ESM.pdf]

**A**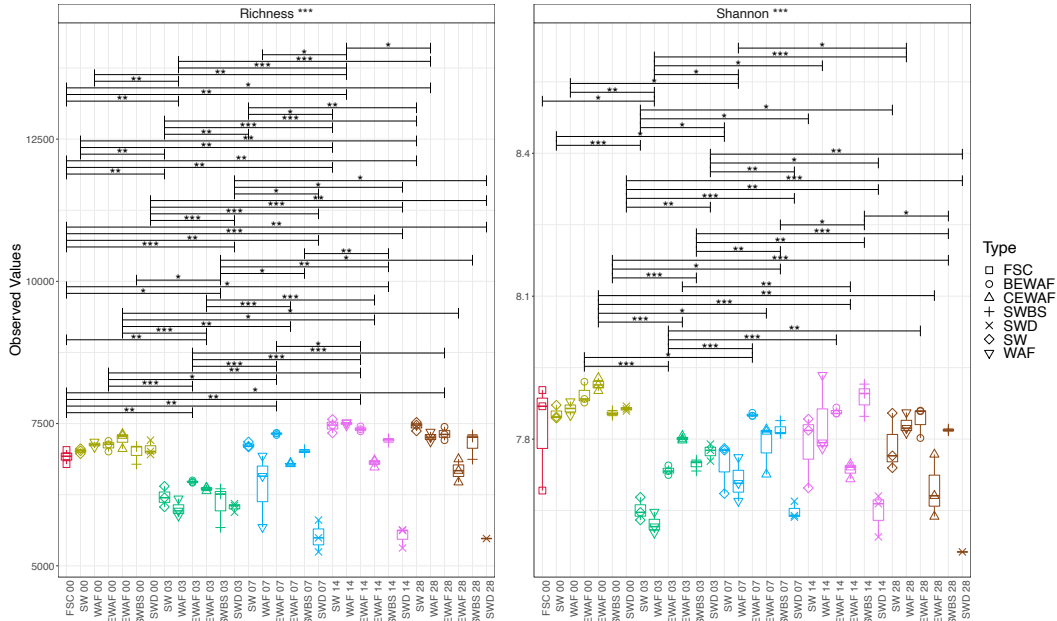**B**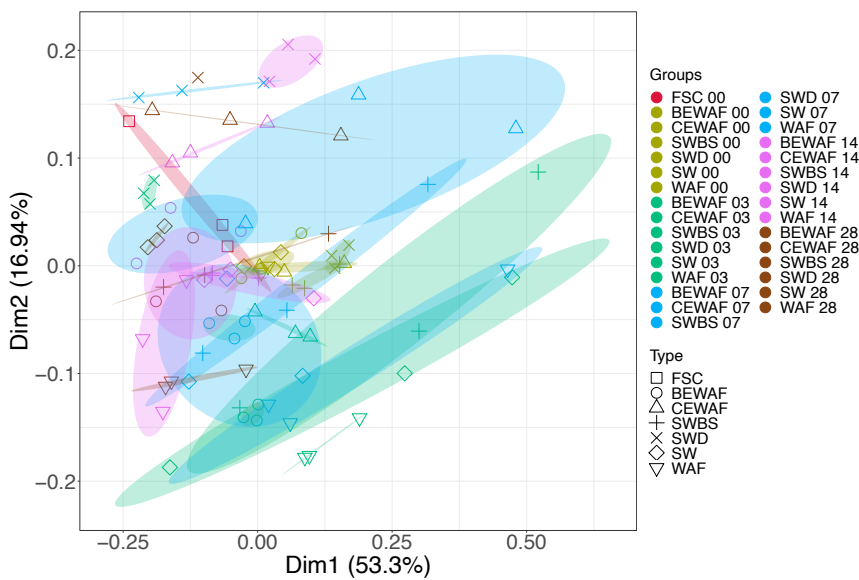

Supplement: Supplementary file 8 — Additional file 7: Supplementary Figure S4. (A) Predicted functional alpha diversity of microbial pathways (expressed as number of KEGG orthologs). Statistically different treatments (pair-wise ANOVA) are connected by bracket and the level of significance is shown with: *(p < 0.05), ** (p < 0.01), or *** (p < 0.001). (B) Principal coordinate analysis (PCoA) on beta diversity measured with Bray-Curtis dissimilarity distance matrix. In both (A) and (B) treatments are represented by shape (shown on graph) and incubation time by colour: red – baseline microbial community at time of seawater sampling, olive green – day 0, green – day 3, blue – day 7, pink – day 14, and brown – day 28. FSC is the in-situ baseline microbial community, WAF - seawater and oil only, BEWAF – seawater, crude oil and biosurfactant, CEWAF – seawater, crude oil and synthetic dispersant, SW - seawater only, SWBS - seawater and biosurfactant, and SWD – seawater and synthetic dispersant. [file 40168_2021_1143_MOESM8_ESM.pdf]

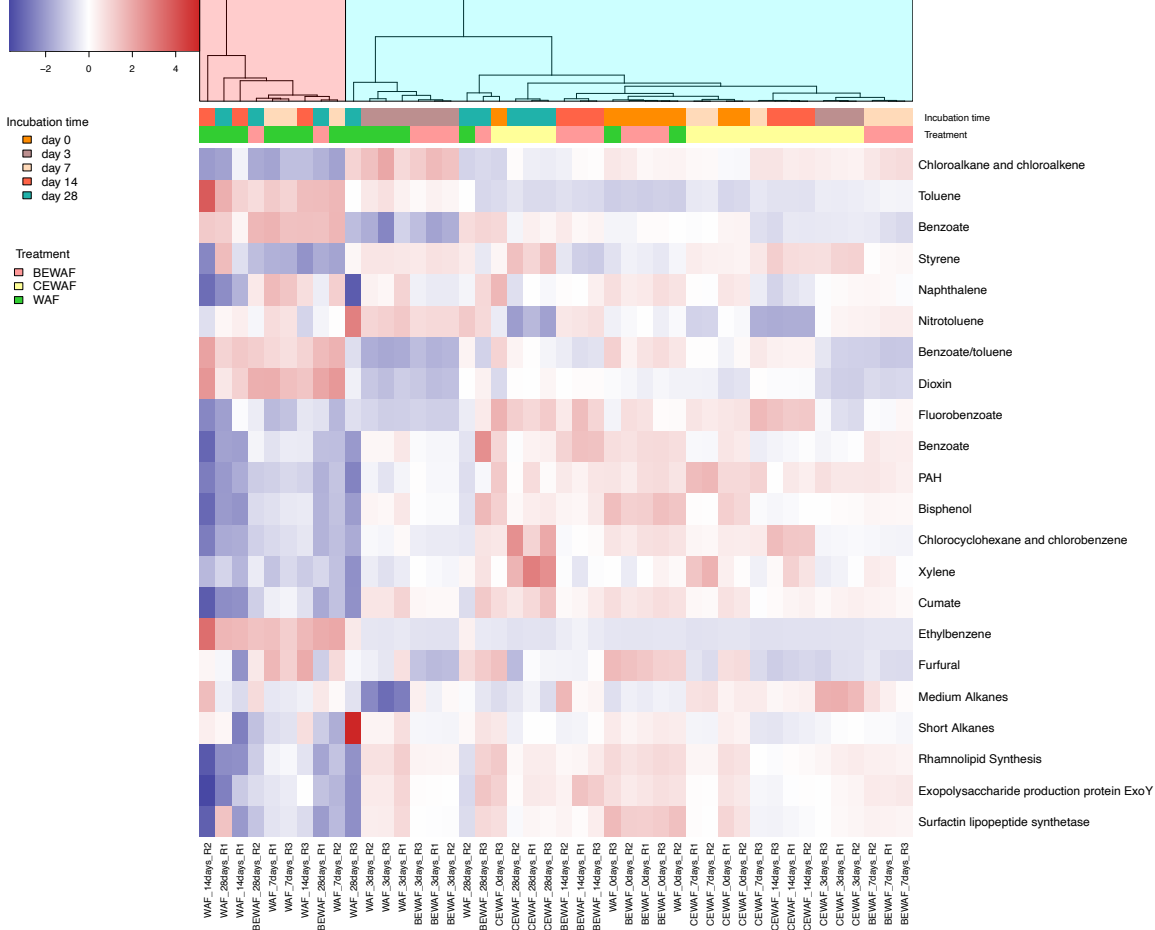

Supplement: Supplementary file 9 — Additional file 8: Supplementary Figure S5. Heatmap showing the scaled log abundance (color key on top left) of aliphatic and aromatic degradation, and biosurfactant synthesis pathways. Pathways are shown along the y-axis and BEWAF (seawater, crude oil, and biosurfactant), CEWAF (seawater, crude oil, and synthetic dispersant), and WAF (seawater and crude oil only) samples along the x-axis. The two color-coded bars on top of the heatmap indicate their treatments and incubation days status. Hierarchical clustering of the samples (top) is based on the correlation between samples’ predicted gene expression. [file 40168_2021_1143_MOESM9_ESM.pdf]

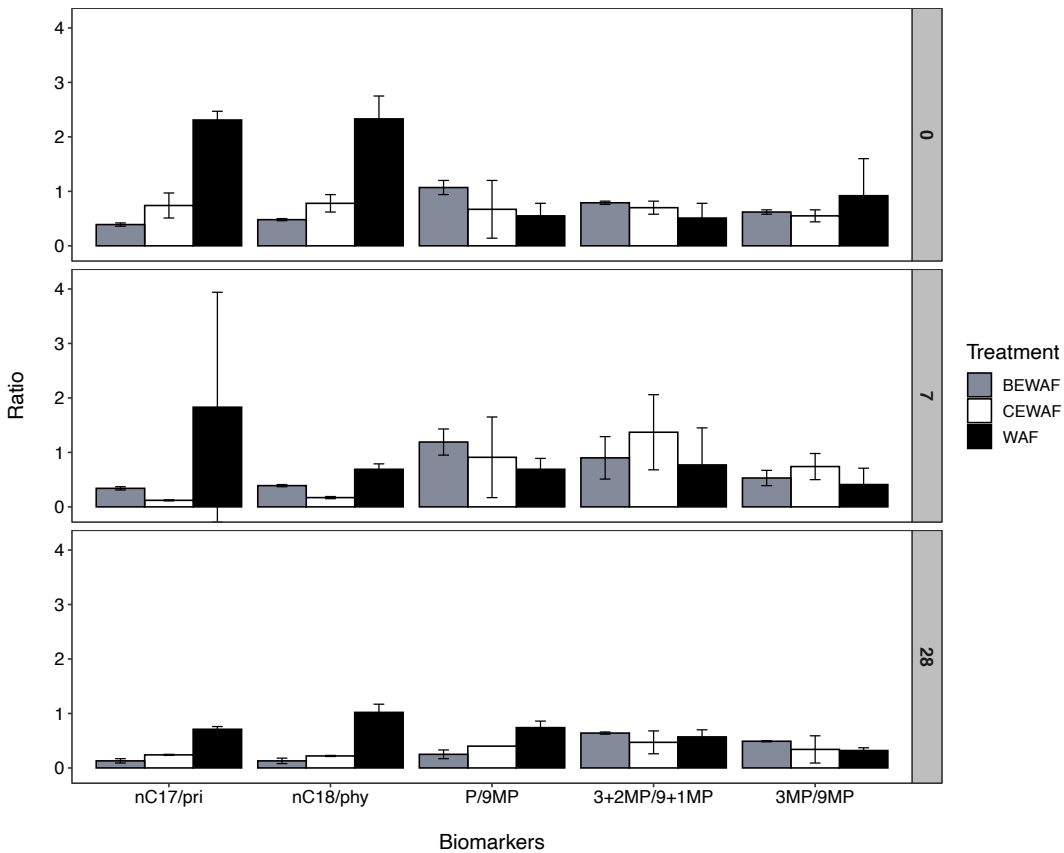

Supplement: Supplementary file 10 — Additional file 9: Supplementary Figure S6. Differences in aliphatic and polycyclic aromatic hydrocarbon biomarker ratios of three different treatments amended with crude oil: BEWAF (seawater, crude oil, and biosurfactant), CEWAF (seawater, crude oil, and synthetic dispersant), and WAF (seawater and crude oil only), over time in days (grey boxes): nC17/pristane (nC17/pri), nC18/phytane (nC18/phy), Phenanthrene/9-methylphenanthrene (P/9MP), (3+2)-methylphenanthrene/(9+1)-mehylphenanthrene (3+2MP/9+1MP), and 3-methylphenanthrene/9-methylphenanthrene (3MP/9MP). Values are the mean of three independent replicates (except for BEWAF day 0 (two replicates) and CEWAF day 28 (one replicate)) +/- standard deviation. [file 40168_2021_1143_MOESM10_ESM.pdf]
