# Supplementary material for: Response and oil degradation activities of a northeast Atlantic bacterial community to biogenic and synthetic surfactants
Source: Microbiome. 2021 Sep 21;9:191. doi: 10.1186/s40168-021-01143-5 (PMC8456599; doi:10.1186/s40168-021-01143-5)
Supplement: Supplementary file 5 — Additional file 4: Supplementary Figure S2. Summary of significant predictive parameters (i.e., treatments and incubation days) determined by regression analysis based on unsupervised machine learning for two alpha diversity measures (Richness and Shannon index), NTI, NRI, and Local contribution to beta diversity (LCBD). The parameters shown here are from the optimal model for each metric, where blue represent negatively and red – positively influencing variables, respectively. FSC is the in-situ baseline microbial community, WAF - seawater and oil only, BEWAF – seawater, crude oil and biosurfactant, CEWAF – seawater, crude oil and synthetic dispersant, SW - seawater only, SWBS - seawater and biosurfactant, and SWD – seawater and synthetic dispersant. [file 40168_2021_1143_MOESM5_ESM.pdf]

	Richness	Shannon	NRI	NTI	LCBD Bray-Curtis	LCBD Unweighted UniFrac	LCBD Weighted UniFrac
Treatment_CEWAF	***		***		***		***
Treatment_SWD	***	**	***	*	***	***	***
Treatment_BEWAF		**	***				
Treatment_SWBS							
Treatment_SW				*	***	**	
Treatment_WAF			*		***		
Treatment_FSC	***			**	***	***	***
Incubation_day_0	***	***	***		***		
Incubation_day_3	***	**	**			***	
Incubation_day_7	***			**			
Incubation_day_14					***		**
Incubation_day_28					***	***	***
