# Supplementary material for: Response and oil degradation activities of a northeast Atlantic bacterial community to biogenic and synthetic surfactants
Source: Microbiome. 2021 Sep 21;9:191. doi: 10.1186/s40168-021-01143-5 (PMC8456599; doi:10.1186/s40168-021-01143-5)
Supplement: Supplementary file 7 — Additional file 6: Supplementary Figure S3. Differential heat trees showing the key (significant) differential taxa (DESeq2; Wilcoxon p-value test adjusted with multiple comparison) in seawater-only control treatment (SW). The top 3 subsets with the highest correlation with the full ASV table considering Bray-Curtis distance (PERMANOVA) are listed for each treatment. The grey tree is the taxonomy key for the smaller unlabelled coloured trees. The colour of each taxon represents the log-10 ratio of median proportions of reads observed in each treatment. The size of tree nodes shows the number of ASVs (here labelled as OTUs) present in each sample. [file 40168_2021_1143_MOESM7_ESM.pdf]

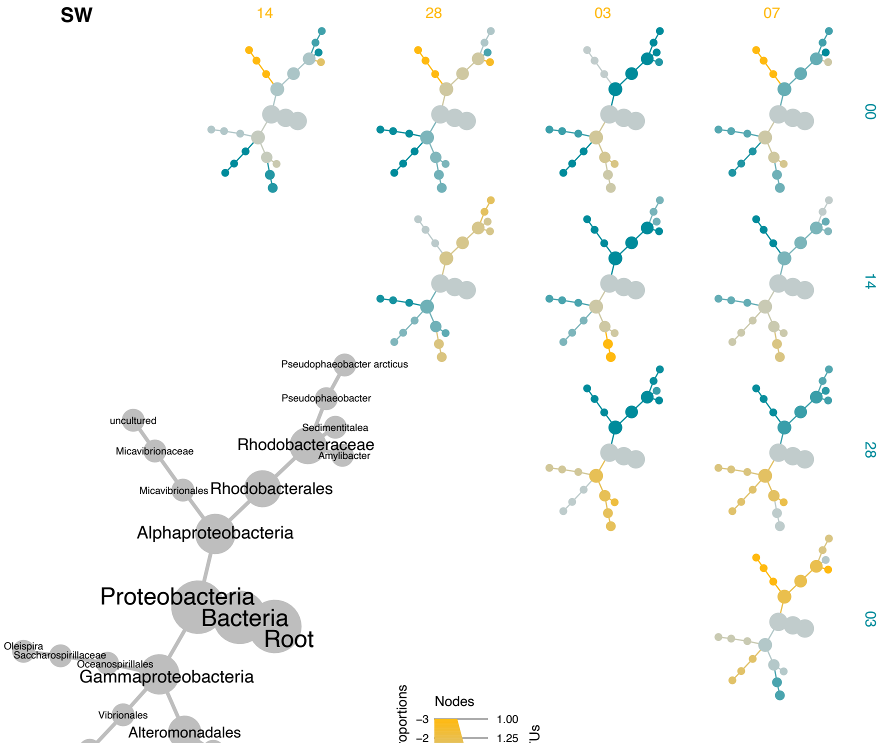

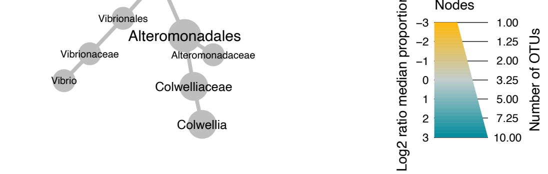

No	Subset	Correlation with full ASV table	PERMANOVA
1	*Alteromonadales_unclassified + Pseudophaeobacter + Sedimentitalea + Colwellia + Oleispira + Amylibacter + Colwellia + Vibrio + Rhodobacteraceae_unclassified + Micavibrionaceae_uncultured*	0.950	$R^2 = 0.162$<br>P = 0.054
2	*Alteromonadales_unclassified + Pseudophaeobacter + Sedimentitalea + Colwellia + Oleispira +Amylibacter + Colwellia + Vibrio + Rhodobacteraceae_unclassified*	0.946	$R^2 = 0.159$<br>P = 0.068
3	*Alteromonadales_unclassified + Pseudophaeobacter + Sedimentitalea + Cowellia + Oleispira + Amylibacter + Vibrio + Rhodobacteraceae_unclassified*	0.937	$R^2 = 0.145$<br>P = 0.097
